# Small but mighty: a functional look at bacterial sHSPs

**DOI:** 10.1007/s12192-020-01094-0

**Published:** 2020-04-16

**Authors:** Igor Obuchowski, Krzysztof Liberek

**Affiliations:** Intercollegiate Faculty of Biotechnology UG-MUG, University of Gdansk, Abrahama 58, 80-307 Gdansk, Poland

**Keywords:** Small HSPs, Bacteria, Proteostasis, Chaperone, Aggregation

## Abstract

Small heat shock proteins (sHSPs) are widespread in every kingdom of life, being indispensable for protein quality control networks. Alongside canonical chaperone functions, sHSPs seem to have been a very plastic scaffold for acquiring multiple related functions across evolution. This review aims to summarize what is known about sHSPs functioning in the Bacteria Kingdom.

## From discovery to common features

The first discovered member of the small heat shock protein family was α-crystallin, which has been known for more than a century to be the major structural protein of the vertebrate eye lens (Mӧrner 1894). Many years later, a well-established family of α-crystallins was found to be related to *Drosophila melanogaster* small heat shock proteins (abbreviated as sHSPs), which justified including them in a common classification group of sHSPs (Ingolia and Craig 1982). This, together with the heat shock response-focused research boom of these years, has led to broad investigations on small heat shock proteins.

Although sHSPs sequence conservation is rather limited, especially in contrast to other heat shock proteins, they started to be identified by homology to α-crystallins and the fruit fly sHSPs (Key et al. 1981; Russnak et al. 1983; Booth et al. 1988; Nerland et al. 1988; Verbon et al. 1992; Lee et al. 1992). As the number of identified sHSPs has been growing, their polypeptides were found to be typically subdivided into an α-crystallin domain, the most homologous region in their sequence and a much less conserved flanking N- and C-termini (Van Montfort et al. 2001). At the same time, multiple sHSPs were shown to form large dynamic complexes (Arrigo and Welch 1987; Behlke et al. 1991) and, later, their dissociation to be regulated by temperature changes (Fu and Chang 2004). Accompanying structural studies has shown that sHSPs are especially rich in β-structures (Li and Spector 1974; Merck et al. 1993), finally leading to the first resolved bacterial sHSP structure (Kim et al. 1998a, b). It was later found that the β-sandwich fold is a common, highly conserved feature of all sHSPs.

In addition to structural studies, a great deal of effort was put into determining the functional traits of sHSPs. Initial studies on α-crystallins focused on the medical contexts, especially prevention of cataract formation (Roy and Spector 1976) and later on roles in tumor cell growth (Gaestel et al. 1989) and cell differentiation (Stahl et al. 1992). Concerning the presence of sHSPs in organisms from every kingdom of life, it appeared challenging to elucidate their common functions (Schlesinger 1986) suggested by their striking fold conservation. sHSPs were often found to be highly overexpressed at heat stress—conferring thermotolerance to some organisms (Loomis and Wheeler 1982; Berger and Woodward 1983)—but showing no feasible phenotype when disrupted in others (Susek and Lindquist 1989; Praekelt and Meacock 1990). It took detailed biochemical studies to demonstrate that sHSPs act as molecular chaperones both in animals (Jakob et al. 1993; Wang and Spector 1995), plants (Lee et al. 1995) and bacteria (Chang et al. 1996; Thomas and Baneyx 1998).

From the evolutionary point of view, small HSPs divergence across all kingdoms of life seems to have been driven with great plasticity towards acquiring different functions (De Jong et al. 1993; Carra et al. 2017). There is a clear tendency to increase the number of sHSP-coding genes with increasing organism complexity, which accompanies an increased diversity of performed functions. Bacteria, considered to be the least complex organisms, usually express only one or two sHSPs (Haslbeck et al. 2005) that can still perform several functions in the bacterial cell. To date, the most important reported bacterial sHSPs functions are (i) molecular chaperone function, which can be subdivided into two distinct modes of action, transient interactions with unfolding polypeptides at mild proteotoxic stress and high-affinity interactions observed at massive aggregation events; (ii) protection of cell membrane components and membrane integrity; and (iii) a handful of more specific functions dedicated to survival in adverse environments.

## Chaperone activity: stable interactions

The best studied chaperone activity of sHSPs is preventing formation of large amorphous aggregates, which seems to be evolutionarily the oldest and most important sHSPs function for bacteria. To perform it, sHSPs intercept unfolding intermediates and co-assemble in so-called sHSPs-substrate assemblies that serve as the safe storage for polypeptides before refolding. This section will focus on formation, architecture, and refolding of substrates from sHSPs-substrate assemblies.

In 1996, after multiple evidence appeared of eukaryotic sHSPs being molecular chaperones (Jakob et al. 1993; Boyle and Takemoto 1994; Singh et al. 1995; Wang and Spector 1995; Raman et al. 1995), Hsp16.3 from *Mycobacterium tuberculosis* was shown to suppress citrate synthase (CS) thermal aggregation, although it could not protect CS activity nor refold it afterwards (Chang et al. 1996). The same year *Escherichia coli* IbpA & IbpB, previously described as inclusion body associated proteins (Allen et al. 1992), were found to co-localize with the aggregated protein fraction in heat shock conditions (Laskowska et al. 1996). Later, they were also shown to confer thermotolerance and to stabilize aggregated proteins for further refolding (Thomas and Baneyx 1998; Veinger et al. 1998). These observations, taken together with sequence homology to eukaryotic sHSPs, gave a solid proof for considering bacterial sHSPs as molecular chaperones.

sHSPs chaperone activity is exerted by stabilization of structurally damaged proteins for subsequent refolding by the Hsp70-Hsp100 bi-chaperone system. It is achieved by sHSPs binding to partially unfolded polypeptides in stress conditions and by driving their aggregation towards characteristic complexes called sHSPs-substrate assemblies. sHSPs showing this activity are often called aggregases, which might be misleading as sHSP-substrate assemblies and the assembly process itself differ from amorphous aggregates and aggregation. To date, the direct molecular mechanism of the assembly formation process remains elusive except in several details. It is known that substrate binding, executed by the N-terminus and α-crystalline domain (Fu et al. 2005; Tomoyasu et al. 2010; Fu et al. 2013b; Fu and Chang 2006), is preceded by sHSPs oligomers dissociation into smaller species—preferably dimers (Fu and Chang 2004)—that are postulated to be the active species in this process. It is typically observed also for nonbacterial sHSPs (Haslbeck and Vierling 2015).

Efficient polypeptides sequestering in assemblies requires the presence of stoichiometric amounts of sHSPs—at least in vitro (Friedrich et al. 2004; Fu and Chang 2004; Jiao et al. 2005). In vivo sHSP genes are commonly found to undergo massive expression upregulation in stress conditions. It is more pronounced than upregulation of any other chaperone as judged by cellular protein content and transcription profiling (Richmond et al. 1999; Münchbach et al. 1999; Lee et al. 1998)—presumably providing enough sHSPs for efficient in vivo substrate sequestering. On the other hand, little is known of the substrates trapped by bacterial sHSPs. It can only be deduced from studies on yeast sHSPs, that these are stored in near-native conformation (Ungelenk et al. 2016), which is probably one of the factors facilitating further disaggregation and refolding. Speaking of assemblies architecture, substrate molecules are postulated to be held in the core of the assembly with only a limited number of sHSPs and shielded from the environment by the sHSPs outer shell (Żwirowski et al. 2017); however, there are no direct structural data on this subject.

The ability of sHSPs to stabilize unfolded polypeptides provoked obvious concerns about the later fate of trapped polypeptides. In 1998, Veigner and colleagues showed that *E. coli* IbpB, when present during malate dehydrogenase thermal aggregation, improves further disaggregation by dedicated chaperones (Veinger et al. 1998). This finding, which was also established for eukaryotic sHSPs (Lee et al. 1997), has led to the integration of sHSPs as a part of chaperone network. It became clear that bacterial (and other) sHSPs modulate protein aggregation in order to hold unfolded polypeptides in a refolding competent state (Matuszewska et al. 2005; Ratajczak et al. 2009). In 2017, Żwirowski et al. proposed the mechanism of extraction and refolding of misfolded polypeptides from sHSPs-substrate assemblies. They have shown that specifically Hsp70 chaperone binds to the assemblies to outcompete sHSPs, which allows for single polypeptides extraction by recruited Hsp100 disaggregase. The authors suggested a lack of direct interaction between Hsp70 and sHSPs being removed from assemblies—just affinity competition for unfolded polypeptides trapped in assemblies. Additionally, several experiments were performed with yeast proteins, suggesting a common described mechanism (Żwirowski et al. 2017).

To date, the majority of studies linking sHSPs action in protein aggregation and their interference with disaggregating chaperones were carried out in the *E. coli* system, where 2 sHSPs—IbpA and IbpB—cooperate with each other. Interestingly, IbpA, when present during substrate aggregation, is enough to generate assemblies with the substrate but also inhibits its further disaggregation. This inhibition is lost in the presence of IbpB alongside IbpA. However, IbpB alone has a much less pronounced effect on disaggregation, being barely able to generate assemblies with unfolding substrate (Ratajczak et al. 2009; Matuszewska et al. 2005; Thomas and Baneyx 1998). Recently, IbpB activity was shown to interfere with IbpA canonical substrate binding, which results in easier sHSPs removal from assemblies and effective disaggregation (Obuchowski et al. 2019).

An alternative example of 2 sHSPs bacterial system comes from *Deinococcus radiodurans*, where sHSPs act separately. One of them, Hsp20.2, is  very effective in assembly generation when in the presence of an aggregating substrate, and the other, Hsp17.7, is capable of sustaining substrate activity (or postponing activity loss) in otherwise denaturing conditions (Bepperling et al. 2012). This activity protection is achieved by transient interactions with the substrate, which will be discussed as a stand-alone phenomenon in the next section. Overall, both *E. coli* and *D. radiodurans* systems seem rather atypical as most bacteria express only one sHSP (Haslbeck et al. 2005). The best studied single sHSP is Hsp16.3 from *M. tuberculosis* that was used for functional studies showing typical assembly forming chaperone activity (Chang et al. 1996), surface hydrophobicity changes (Yang et al. 1999), and oligomers dissociation (Fu and Chang 2004) upon heat treatment. Intensive studies on Hsp16.3 have also revealed its non-chaperone functions that will be described in another section.

## Chaperone activity: transient interactions

Another important, yet less studied, example of sHSPs chaperone activity is their ability to protect enzymes from inactivation or postpone their activity loss upon mild denaturing conditions. It is exerted *via* transient, cyclic interactions (in contrast to stable assembly generation-driving interactions) with hydrophobic regions of slightly damaged protein substrates, somehow directing them back to a native fold. This section will focus on several bacterial sHSPs that were shown to act in this mode of chaperone activity.

From the mechanistic point of view, it is highly elusive how bacterial sHSPs protect enzyme activity; however, it can be deduced from several studies on vertebrate sHSPs. These have been shown to weakly and transiently interact with misfolded intermediates—forming dynamic high molecular weight assemblies (Kulig and Ecroyd 2012). Target substrate hydrophobicity and stability largely determines if sHSPs would tightly interact with the substrate, stabilizing it for further refolding or transiently binding and release. As the misfolding intermediate is subsequently bound and released, it is secured from aggregation and can fold into the native structure (Kulig and Ecroyd 2012; Hatters et al. 2001).

In bacteria, an enzyme activity protection assay was initially applied for *M. tuberculosis* Hsp16.3. Although the assay showed chaperone activity towards citrate synthase as monitored by static light scattering, the authors could not observe any protection of citrate synthase activity (Chang et al. 1996). On the other hand, *E. coli* IbpA and IbpB turned out to be more successful in this type of experiment. Together, they were shown to protect luciferase from thermal inactivation (although weakly) (Matuszewska et al. 2005) and both separately and together—to protect several other enzymes from oxidative and freeze-thaw inactivation (Kitagawa et al. 2002). Interestingly, the authors claim IbpB to be more effective than IbpA in enzyme activity protection (Kitagawa et al. 2002), which is consistent with later reported in vivo IbpB ability to protect metabolic enzymes activities during heat stress (Fu et al. 2013a). In contrast, IbpA was shown to be much more potent than IbpB in forming stable assemblies with aggregating polypeptides. This suggests a diversity in their activities although they cooperate when acting as mixed complexes (Ratajczak et al. 2009; Matuszewska et al. 2005; Hochberg et al. 2018; Obuchowski et al. 2019). Finally, it seems that cooperation is not a key feature for this type of sHSPs activity. Here is an example from *D. radiodurans*, which Hsp17.7 was shown to effectively protect CS from thermal inactivation in contrast to its paralog Hsp20.2 that can neither protect CS activity nor cooperate with Hsp17.7 (Bepperling et al. 2012).

Leaving aside the cooperation issues, it seems that the ability to form assemblies with aggregating substrates and protect enzyme activity are somehow in opposition. This is supported by research on single sHSPs: *M. tuberculosis* Hsp16.3 that is only able to form assemblies (Chang et al. 1996) and—in contrast—its close relative sHsp18 from *Mycobacterium leprae*, which effectively protects restriction enzymes from thermal inactivation, however was not assayed for generating stable complexes/assemblies with aggregating substrate (Lini et al. 2008).

## Chaperone activity: conclusion

Although sHSPs-dependent enzyme protection and sHSPs-substrate complex formation were already shown in the very first publication attributing sHSPs with chaperone activity (Jakob et al. 1993), these modes of action are rarely assayed when new bacterial sHSP appears. The most exploited assay in this field (and technically the easiest) is in vitro aggregation protection monitored *via* static light scattering. It does not directly indicate whether prevention of sample scattering increase is achieved by protecting a substrate’s native fold or by scavenging unfolded polypeptides within soluble assemblies. Therefore, it does not allow discrimination of sHSPs chaperone activity modes. The same concern may apply to widely exploited experiments on in vivo aggregation, where the amount of cellular insoluble (aggregated) protein is compared between strains. Here again, one could ask if aggregates volume is lower due to sHSPs-dependent substrate activity (fold) maintenance or by providing a more potent substrate for effective disaggregation (sHSPs-substrate assemblies instead of amorphous aggregates).

From the “end user” point of view, however, it is the final outcome that matters, e.g., reduced aggregation/aggregates volume. Thus, we can see two different strategies to achieve that. It can only be speculated that the path of aggregation modification may be more effective in counteracting severe proteotoxic stress when unfolding events occur frequently and rapidly among a larger pool of polypeptides. In contrast, the activity protection path might be favorable under less severe conditions, when only a smaller pool of less stable substrates is exposed to the risk of unfolding.

Finally, there is an evidence for the third option. Klein and colleagues (Klein et al. 2001) have shown that IbpA, a single sHSP from the marine bacterium *Vibrio harveyi*, complexes in vivo with aggregated protein fraction similarly to most typical sHSPs. However, one of their experiments suggests that the aggregated protein fraction (containing IbpA) is highly stable during cell recovery. This in turn could suggest a dilution of IbpA-detoxified, stable aggregate species (IbpA-substrate assemblies?) by cell divisions as a mechanism of aggregates handling in *V. harveyi*; however, this would require further research to prove.

## Membrane-focused chaperone

Among chaperone-focused research, accompanying localization studies have shown several bacterial sHSPs to locate in cell membranes (Lee et al. 1992; Laskowska et al. 1996; Otani et al. 2005). This feature was further exploited in several different bacteria species, giving rise to the concept of sHSPs as lipochaperones (Maitre et al. 2014). This section will attempt to summarize what is known about sHSPs-membranes relationship.

Starting from the work of Horvath and colleagues, who identified *hsp17* as a “fluidity gene” in *Synechocystis* PCC 6803 (Horvath et al. 1998), it was shown that several sHSPs are capable of reducing membranes fluidity in permissive or heat stress conditions and in the presence of organic solvents (Torok et al. 2001; Capozzi et al. 2011). To perform this task, sHSPs associate with membranes not as higher-order oligomers, but rather in the form of dissociated species (Zhang et al. 2005; Maitre et al. 2012), which are also active substrate-binding forms concerning canonical chaperone activity. Subsequently, Maitre and colleagues proposed a combined model of chaperone and lipochaperone *Oenococcus oeni* Lo18 activities that summed up extensive studies in this field (Maitre et al. 2014). However, no further molecular details of Lo18 lipochaperone sHSPs activity are available.

Moving from general to more specific lipochaperone activity, an ability of cyanobacteria sHSPs to maintain thylakoid membrane integrity and their canonical chaperone activity towards phycocyanins (Nakamoto and Honma 2006) were integrated and analyzed in the context of resistance to UVB-induced damage (Balogi et al. 2008) and oxidative stress (Sakthivel et al. 2009) in *Synechocystis*. Presented data strongly highlight the importance of HspA, cyanobacterial sHSP, for preserving photosynthetic thylakoid functions—both through maintaining general thylakoid membrane integrity (lipochaperone) and by specific protection of phycobilisomes and PSII complexes from inactivation (dedicated chaperone).

## Chaperone for special tasks

Small HSPs are featured with the least conserved sequences among all chaperone families, which allowed great evolutionary plasticity towards acquiring new functions. This is especially evident in multiple sHSPs-expressing organisms like plants or animals, where many sHSPs perform other than general chaperone functions. In bacteria, it is less evident (or less investigated), although there are several 'case studies' that demonstrate specific, dedicated chaperone-target interactions or other non-chaperone sHSP function. This section is an attempt to briefly summarize bacterial sHSPs activities that are separate from the already discussed canonical chaperone activities.

An example of specific sHSP function was found in *Agrobacterium tumefaciens*. HspL, one of the four sHSPs expressed in this bacterium, was found to be important for *A. tumefaciens* virulence towards plant cells, e.g., transferring its DNA into plant cells in order to take over plant metabolism. HspL, but no other *A. tumefaciens* sHSP, effectively protects VirB8 protein (Tsai et al. 2012) that is an essential assembly factor for type IV secretion system responsible for DNA injection into plant cells (Baron and Cellulaire 2006). VirB8 protein is conserved across evolution (Baron and Cellulaire 2006); therefore, a follow-up study, concerning the VirB8-dependent mammal pathogen, appeared. *Brucella suis*, a facultative intracellular bacterial pathogen of mammals, also uses the IV secretion system for virulence particle delivery to the host cells. Unlike in *A. tumefaciens*, it was shown that *B. suis* IbpA is not required for virulence (Berta et al. 2014). The second *B. suis* sHSP (also annotated as IbpA) was not analyzed based on significantly lower homology to *A. tumefaciens* HspL (Berta et al. 2014). Considering these data, it seems that the relationship of VirB8 to HspL found in *A. tumefaciens* is rather limited to a narrow species group.

As sHSPs are responsible for protecting proteostasis on the molecular level, they may also play a more general role in some of the strategies for survival in a hostile environment. One of these strategies is a biofilm formation that is a three-dimensional, complex structure formed of bacteria settled in an extracellular matrix. Biofilms are more resistant to various stresses (antibiotics, heavy metal ions, oxidation) than free-living bacterial cells, enhancing their ability to survive (Flemming et al. 2016). In *E. coli*, it was shown that IbpA and IbpB indirectly influence biofilm formation, delaying its establishing when absent. In *ΔibpAB* strains, cells are affected by endogenous oxidative stress, which results in overproduction of indole, that in turn inhibits formation of the biofilm (Kuczyńska-Wiśnik et al. 2010).

Another strategy for survival in adverse environments, where sHSPs may interfere, is cyst formation. In opposition to biofilm, a microbial cyst is a resting/dormant stage, dedicated to passively survive harsh conditions. *Azotobacter vinelandii* is a free-living soil bacterium whose sHSP, Hsp20, was shown to be essential for cyst desiccation resistance. Consistent with the function, *hsp20* gene in *A. vinelandii* is under the control of RpoS sigma factor (Cocotl-Yañez et al. 2014) that governs expression of many genes crucial for bacterial survival in adverse environments. This is however atypical for sHSPs as most of them in related bacterial species are under the control of RpoH paralogs—master regulators of the heat shock response in these species (Tilly et al. 1986).

The most complete story in terms of sHSPs-affected survival in adverse environments comes from *M. tuberculosis*. Its Hsp16.3 sHSP is associated with dormancy and stationary phase, where it was shown to be expressed the most. Hsp16.3 expression results in lower cell susceptibility to autolysis at the cost of slower growth rate (Yuan et al. 1996). Hsp16.3 was also shown to be instrumental in cell wall thickening that provides additional protection during dormancy (Cunningham and Spreadbury 1998). Similarly to the growth on media, research conducted in pathogen-host systems has also shown that Hsp16.3 plays a role in slowing the growth of *M. tuberculosis* during infection (Hu et al. 2006)—being important for TB-characteristic infection latency. Hsp16.3 is also highly induced upon entry into macrophages and is crucial for both pathogen survival and virulence in the host organism (Yuan et al. 2002; Hu et al. 2006).

## Concluding remarks

Bacterial sHSPs as a group are very plastic in approaching protein misfolding, aggregation, and other issues. There seems to be much specialization in their activities, though. Despite abilities to perform completely different tasks, sHSPs unite in adverse environment conditions survival, providing proteostasis protection, virulence, or other, sometimes elusive advantages. Most of these impacts and functions were found to rely on direct, stabilizing contacts with other proteins, membranes, and complexes, indicating the functional origin of sHSPs as chaperones.
